# Current prevalence status of gastric cancer and recent studies on the roles of circular RNAs and methods used to investigate circular RNAs

**DOI:** 10.1186/s11658-019-0178-5

**Published:** 2019-08-16

**Authors:** Fei Jiang, Xiaobing Shen

**Affiliations:** 1 Key Laboratory of Environmental Medical Engineering and Education Ministry, Nanjing Public Health College, Southeast University, Nanjing, 210000 China; 2 Department of Preventive Medicine, Nanjing Public Health College, Southeast University, Nanjing, 210000 China

**Keywords:** Gastric cancer, Prevalence situation, CircRNA, Functions, Laboratory detection methods

## Abstract

Gastric cancer is a malignant tumor with the fifth incidence and third mortality worldwide. There were 951,000 new cases and about 723,000 patients died of it in 2012. Undoubtedly, gastric cancer has been affecting people’s living standards, and is already a major public health problem in China with its population growth and ageing. Even though the detection methods and medical standards have improved, the five-year survival rate of people is still very low. While circular RNA (circRNA) is increasingly attracting attention from researchers, at the same time, its mystery has gradually been uncovered. Many studies have shown that circRNA can act as molecular sponge of miRNA to regulate gene expression and has an obviously different expression profile between cancerous and normal groups, which arouse people’s curiosity and provide new opportunities for early detection of gastric cancer to improve the quality of life of patients. This study reviews current prevalence of gastric cancer in the word and China, as well as the characteristics and functions of circRNA and common laboratory detection methods involving circRNA in gastric cancer.

**Electronic supplementary material:**

The online version of this article (10.1186/s11658-019-0178-5) contains supplementary material, which is available to authorized users.

## Introduction

Gastric cancer (GC) is one of the most serious malignant tumors worldwide with the fifth incidence and third mortality [1], being particularly prevalent in China [2]. Although incidence rates and mortality of gastric cancer are steadily decreasing with improved nutritional compositions and anti-HP antibody used, this disease still poses a huge threat to human health, leading to a poor diagnosis and prognosis for GC patients [3]. The five-year survival rate of people is still very low in patients with serious gastric cancer. The main reason may be lack of high specificity and high sensitivity for early detection while the pathogenesis of the disease is still not fully understood [4].

Therefore, there is a dire need to discover some early detection methods or biomarkers to increase the detection rate of gastric cancer and reduce the incidence and mortality.

CircRNAs are a special class of endogenous noncoding RNAs usually resulting from splicing events or back-splicing events via exon or intron circularization in vitro and vivo experiments [5]. The structure of circRNA is different from linear RNA, with a closed covalent structure [6], which confers vast properties to circRNA which have already been validated [2].

Recently, with the development and improvement of high-throughput sequencing technology and experimental technology, circRNA has been found to occur widely and stably in human, animal and plant cells, even in mammalian tissues [7]. Many studies have shown that circRNA can act as molecular sponge of miRNA to regulate gene expression, tending to explore its relationship with diseases, especially cancer, which also provides new opportunities for early detection of gastric cancer. So, we reviewed the current prevalence of gastric cancer in the word and China, the characteristics and functions of circRNA, and common laboratory detection methods involving circRNA in gastric cancer to give the researchers a general understanding of the circRNA’s characteristics and to promote further study about circRNA with their own conditions. The ultimate goal is to yield a simpler and more effective strategy for the diagnosis and prognosis of gastric cancer through our joint efforts.

## The status of gastric cancer

### Gastric cancer worldwide

**Fig. 1:**
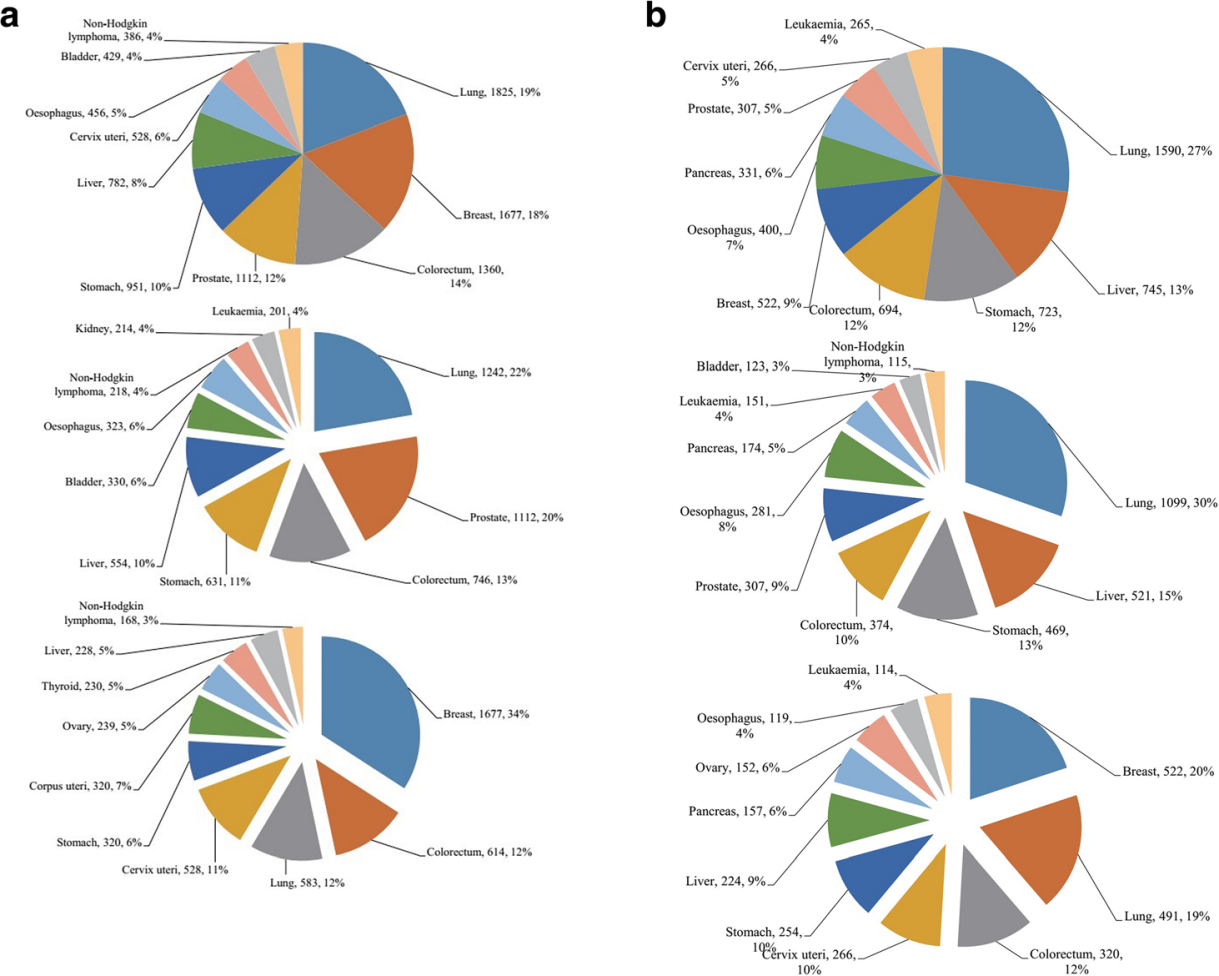
**a** The upper panel shows the top 10 most common types of cancer worldwide (according to new cases) in both sexes—lung, breast, colorectum, prostate, stomach, liver, cervix uteri, esophagus, bladder, and non-Hodgkin’s lymphoma. The middle panel shows the top 10 most common types of cancer worldwide (according to new cases) in males—lung, prostate, colorectum, stomach, liver, bladder, esophagus, non-Hodgkin’s lymphoma, kidney, and leukemia. The lower panel shows the top 10 most common types of cancer worldwide (according to new cases) in females—breast, colorectum, lung, cervix uteri, stomach, corpus uteri, ovarian, thyroid, liver, and non-Hodgkin’s lymphoma. **b** The upper panel shows the top 10 most common types of cancer worldwide (according to number of deaths) in both sexes—lung, liver, stomach, colorectum, breast, esophagus, pancreas, prostate, cervix uteri, and leukemia. The middle panel shows the top 10 most common types of cancer worldwide (according to number of deaths) for males—lung, liver, stomach, colorectum, prostate, esophagus, pancreas, leukemia, bladder, and non-Hodgkin’s lymphoma. The lower panel shows the top 10 most common types of cancer worldwide (according to number of deaths) for females—breast, lung, cervix uteri, stomach, liver, pancreas, ovarian, esophagus, and leukemia

According to the statistics of the International Cancer Research Institute, in 2012, there were 951,000 new cases of gastric cancer worldwide, and about 723,000 patients died of it, which is it the fifth incidence rate (Fig. 1a) and third mortality rate (Fig. 1b) respectively for malignant tumor worldwide. The number of new cases of gastric cancer has changed dramatically compared with the 1975 statistics, when the number was 682,400 [8].

**Fig. 2:**
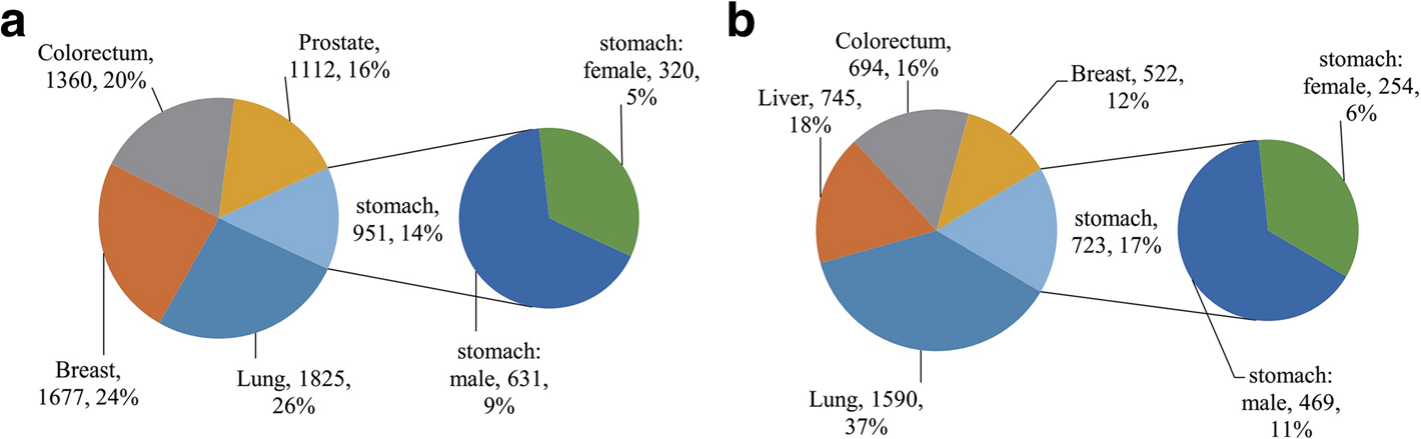
**a** Left. Estimated numbers of new cancer cases globally in 2012 (thousands), with the proportions combined for both sexes. Right. The incidence of new cases stratified by sex. The areas represented in the pie charts are proportional to the number of new cases. **b** Left. Estimated numbers of deaths from cancer globally in 2012 (thousands), with the proportions combined for both sexes. Right. The incidence of death from stomach cancer stratified by sex. The areas represented in the pie charts are proportional to the number of new cases

Figure 1a (upper) show that lung, breast, colorectum, prostate, stomach, liver, cervix uteri, esophagus, bladder, and non-Hodgkin lymphoma are the top ten cancers and the top nine of them represent 64.7% of the global incidence burden in 2012. It is also understood that the top ten new cases of tumors in males are lung, prostate, colorectum, stomach, liver, bladder, esophagus, non-Hodgkin lymphoma, kidney, and leukemia, which are different from females (Fig. 1a middle and below). The cases of stomach cancer in males were nearly twice as numerous as in females (Fig. 2a right). By contrast with the incidence, lung, liver, stomach, colorectum, breast, esophagus, pancreas and prostate combined with cervix uteri represent more than half the mortality burden (68%) worldwide (Fig. 1b upper). Similarly, the top ten deaths from tumors in males are different from females (Fig. 1b middle and below). The cases of stomach cancer in males compared to females also were nearly double (Fig. 2b right).

From the summary of research about cancers, we can obtain the prevalence of GC at the national level. More than half of new cases of gastric cancer occur in developing countries; half occur in East Asia, especially in China and Japan. For mortality, it is still the highest in East Asia. South Asia and Eastern Europe are also relatively high, but North America is the lowest. In a crowd, the incidence of men is nearly twice that of women, wherever [9]. Based on the above finding, we can surmise that the incidence and mortality of GC are region specific [10, 11](Additional file 1: Figure S1).

*H. pylori* is the main risk factor for gastric cancer, involved nearly 90% gastric cancer [12]. According to an epidemiological study, developing countries have a higher prevalence of *H. pylori* infection at all ages [13]. In addition to *H. pylori* infection [14], drinking and smoking are related to the occurrence of gastric cancer. It is also associated with the family history of gastric cancer. Other risk factors include bad eating habits, such as hot food, irregular diet, high salt diet and salty food [15] [16]. We also found some studies that detected the association between genetic polymorphisms and GC, and a genome-wide association study (GWAS) conducted on the basis of the JSNP database for Japanese and Koreans identified two single nucleotide polymorphisms (SNPs) in PSCA (prostate stem cell antigen) [17], which provides us with another direction for studying the high incidence of gastric cancer in East Asia.

### Status of gastric cancer in China

The incidence and mortality rates of cancer in China have been increasing and it has been the main cause of death since 2010, which already is a major public health problem in a country with population growth and ageing [18]. It is undeniable that the aging society is the trend of China’s social population structure, and gastric cancer is an age-related disease [19].

**Fig. 3:**
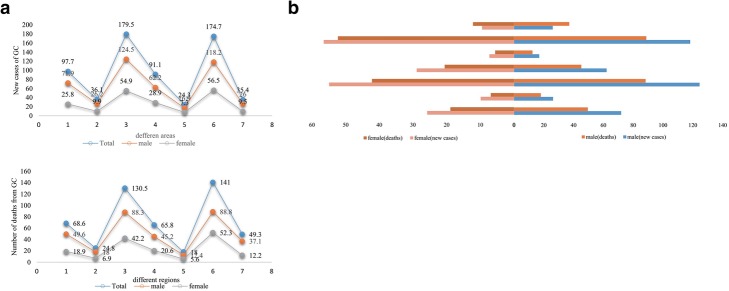
**a** Trends of the new cases of GC (upper panel) and deaths from GC (lower panel) in different regions of China in 2015 (from 1 to 7: North, Northeast, East, Central, South, Southwest, and Northwest China). **b** The upper left and right panels show the number of deaths and number of new cases of cancer in females and males. The lower panel shows the deaths from and new cases of GC in both sexes from Northwest, Southwest, South, Central, East, Northeast, and North China in 2015

The study of Zhang Siwei et al. demonstrated that the number of new cases of gastric cancer in China in 2013 totaled 427,000 and the number of deaths was 301,000. Compared with the estimation results of GLOBOCAN 2012 Data China, the number of cases is slightly higher, the number of deaths is slightly lower, basically the same. However, China incidence rate 21.32/100,000 and mortality rate 14.54/100,000 predicted with world standard were far higher than the prevalence of World Cancer Epidemics (12.1/100,000) and mortality rate (8.9/100,000) predicted by GLOBOCAN [20]. According to the study of Wanqing Chen’s team the numbers of new cases and deaths of east and south China, which are China’s economically developed areas, were more than in other areas (Additional file 2: Figure S2. Figure 3a, b) [10]. The trends in the number of new cases and deaths in these seven places are similar among males and females (Fig. 3a, b). So, it is highly urgent for us to find a reliable and efficient early diagnostic biomarker.

## Origins, properties and functions of circRNA

### Origins of circRNA

CircRNA was discovered as early as the 1970s, In 1976, Sanger et al. obtained a source of viroids from tomatoes and purified the viroid RNA. Hydrodynamic and thermodynamic studies proved that circRNA exists in the viroid [21], which is an earlier research study we discovered that proved the existence of circRNA. But in the next decades, few researchers paid attention to circRNA on account of its lower content or splicing error [22]. Until 1989, T. O. Diener performed plant pathogenic RNAs (viroids and viroid-like satellite RNAs) studies which suggested that circular RNAs may be relics of pre-cellular RNA evolution and indicate that the structure enhanced the survival of RNA [23], which was termed “exon shuffling” or “noncolinear splicing.” With the development and application of sequencing technology, more and more circRNAs have been discovered in animals and human cells [24, 25]. Thomas B Hansen et al. also found circRNA involved in gene regulation in their study – Cerebellar Degeneration-Related protein 1 (CDR1) [26]. Then in 2012, Salzman discovered nearly 80 circular RNAs in human cells due to the application of high-throughput technology. And in 2013, the journal *Nature* published two circRNA research studies in the same period. Since then, circRNA-related research has grown rapidly and has gradually become a new star in the non-coding endogenous RNA field.

### Properties of circRNA

First is the special structure of circRNA. As we mentioned before, the covalently linked ends of circRNA have been found in pathogens such as viroids, satellite viruses [21] and hepatitis delta virus [27], which is 3′ and 5′ joined, called “back splice” [22], different from other lncRNAs. The first detected on back spliced RNAs was on specific genes, where the exons were joined together rather than in linear order [28]. The joining takes place at a flanked site formed by an acceptor splice site and a donor site (head-to-tail) [24].

**Fig. 4:**
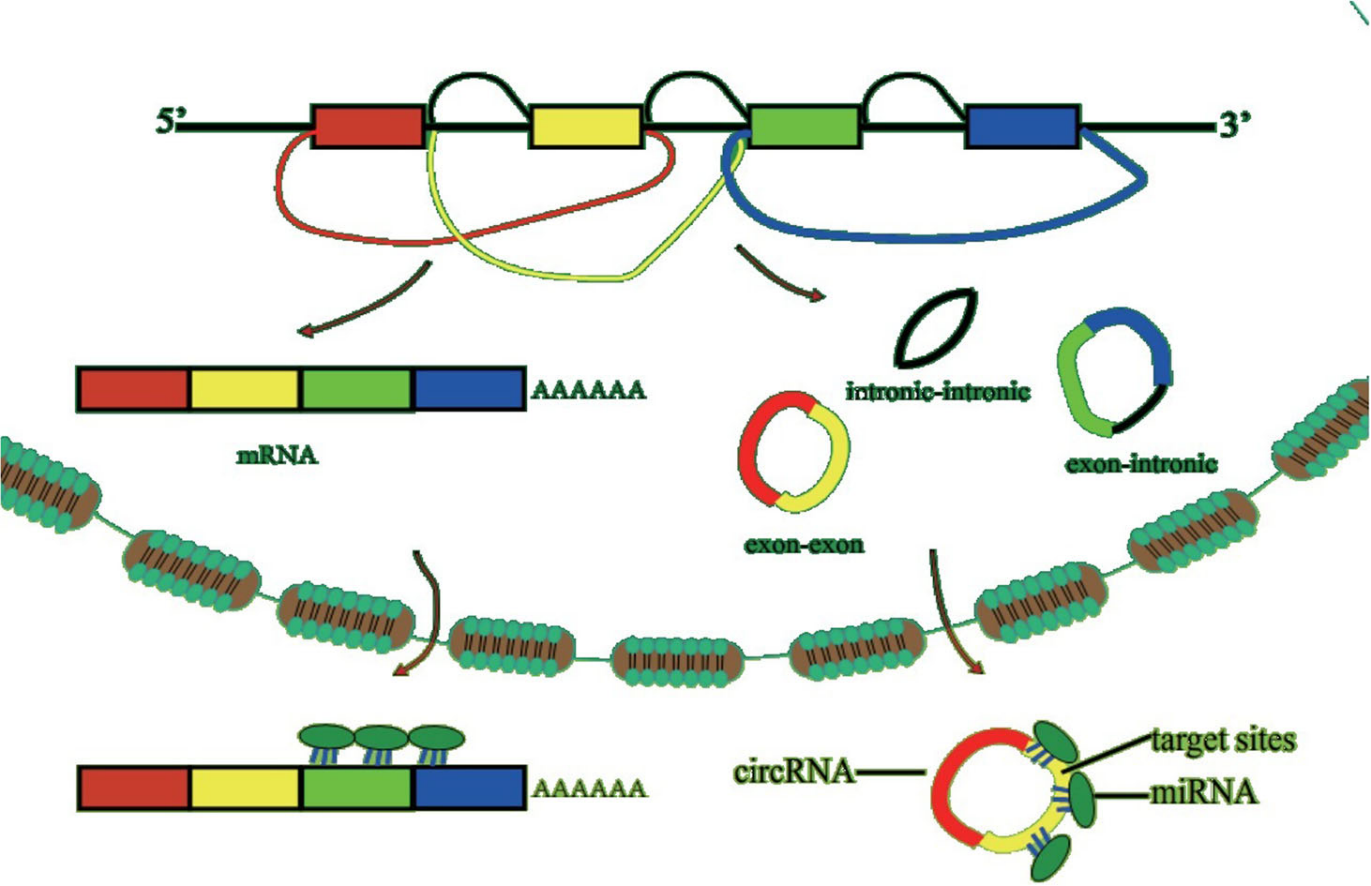
The three forms of covalently joined circRNAs in cells: exon-exon, exon-intron (intronic), and intron-intron (intronic-intronic). circRNAs may serve as miRNA sponges to prevent binding to target genes

There are three forms of back splice, exon-exon [29], intron-intron [30] and exon–intron [31], in covalently joined circRNAs (Fig. 4). The first form is predominantly located in the cytoplasm [1, 22, 25, 32], while the two remaining forms are predominantly located in the nucleus [30, 33]. Therefore they have different functions in biological processes.

Secondly, circRNAs are abundant. Julia Salzman et al. using RNA-Seq found many examples of transcripts where the exon encoded in the genome and the order was a circular permutation in the unrearranged human genome [4]. The further study of Sebastian Memczak et al. found 1950 circRNAs in human cells, 1903 circRNAs in mouse and 724 circRNAs in *C. elegans*. Combined with human leukocyte data and sequencing data, they also found that circRNA has specific expression according to cell type and disease stage [24]. That is to say, more and more circRNAs would be identified by genomic methods and its abundance is more than was expected, as well as its potential function in disease.

Next, circRNAs are stable. For example, William R. Jeck et al. treated Hs68 cells with an inhibitor of transcription, actinomycin D, and total RNA was harvested at indicated time points. While circRNA is very stable in cells, the half-lives of the majority species are over 48 h, compared with the abundant mRNAs, whose half-lives are less than 20 h and proteins less than 3 h [22]. Moreover, they also have high stability in exosomes, blood and other body fluids [34, 35]. Hence, circRNA may be a suitable biomarker in diagnosing cancers.

### CircRNAs as sponges of miRNA

More and more newly discovered functions of circRNAs in diverse cellular processes accelerated the research of circRNA. Some circRNAs could serve as miRNA sponges by sequestering and preventing miRNAs from binding target genes [36] (Fig. 4). Many surveys have found that there are plentiful target sites in circRNAs to absorb miRNAs. Owing to the plentiful target sites, the circRNA was considered as a ‘super-sponge’ for miRNAs. Strikingly, at least 20 miRNA target sites in a single circRNA were found from over 3000 circRNAs, and most of them had an Ago2 binding site [37]. It is understood that numerous annotated back-splicing circRNAs are predominantly localized in the cytoplasm [25]. Also William R. Jeck et al. from their observation found that RNA could target interference circRNAs, which suggested that circRNAs may compete with mRNAs for miRNA binding in the cytoplasm [22].

The best example to support this model is ciRS-7 (circular RNA sponge for miR-7), which is produced by the vertebrate cerebellar degeneration related 1 (CDR1) antisense transcript [38].

### CircRNAs as transcriptional regulators and translation

As described above, there are different structures of circRNAs distributed in the cytoplasm or nucleus, giving them diverse function.

Studies have shown that circRNAs (intron-intron circRNAs) and EIcircRNAs (exon-intron circRNAs) are involved in regulating alternative splicing and transcription, even the expression of parental genes [39, 40]. For example, Cindy Wang Chao et al. knocked out the circRNAs’ back splicing then detected the development of kidney cancer. The back splicing involved a splice acceptor, which is produced from the formin (Fmn) transcript. The formin (Fmn) gene is essential for limb development in mice [41]. They found that limb had an incompletely penetrant renal agenesis phenotype, suggesting that circRNA could regulate transcription. Sebastian Memczak et al. detected CDR1as and miR-7 across mouse brain and tissues and found that CDR1as can act as a post-transcriptional regulator by binding miR-7 in brain tissues. They also obtained the same results with a zebrafish animal model and in vivo [24].

William R Jeck and Norman E Sharpless made an interesting observation that in human fibroblasts: 14% of all exons contain a translation start but 34% of single-exon circles contain a translation start [42]. It suggests that the regulatory function of a single exon may be stronger then exon-intron and intron-intron, also indicating that circRNAs could act as mRNA traps by sequestering the translation start site. Moreover, certain synthetic exon-exon circRNAs have protein-coding capacity both in vivo and in vitro [43]. For example, Yang Yibing et al. discovered that circ-FBXW7 can encode protein, named FBXW7-185aa [32], providing a foundation for circRNAs’ function encoding protein.

### CircRNA’s potential and necessary role in cancers

In colorectal cancer (CRC), circRNA_001569 acts as a miRNA sponge to inhibit the transcription activity of miR-145 and up-regulate miR-145 targets E2F5, BAG4 and FMNL2, promoting the proliferation and invasion of CRC cells [44]. Circular RNA ITCH also has an inhibitory effect on CRC and ESCC (esophageal squamous cell carcinoma) by regulating the Wnt/β-catenin pathway [45, 46]. In OSCC (oral squamous cell carcinoma), circRNA_100290 was capable of regulating the cell cycle and proliferation of OSCC cell lines and the expression profiles were significantly different between the cancer and normal cells [47], which has been proved in many cancers, including gastric cancer. hsa_circ_0000190 [48], circRNA_100269 [49], circular RNA_LARP4 [50], hsa_circ_0014717 [51] and so on, have been verified to be down-expressed in gastric cancer tissues compared with the adjacent normal tissues, which suggests that these circRNAs may be associated with the cancer cell type and disease progression and play a potent and necessary role in cancers. The circRNAs of gastric cancer in recent studies are shown in Table 1. We can also infer that these circRNAs may be used as potential biomarkers in the early diagnosis of gastric cancer.

**Table 1:** The types of circRNAs in gastric cancer (GC) identified in recent studies

CircRNAs	Select reasons	Regulation	Distance from cancerous tissue (cm)	Tissue source	Date (tissue collection)	Fold change	Functions of the studied circRNA	Literature
hsa_circ_0000190	It is down-regulated in GC tissues, and its expression is significantly related to the major clinic pathological factors of patients with GC.	Down-regulated	5	Ningbo Yinzhou People’s Hospital, China	June 2010 to January 2015	–	It is a novel, non-invasive biomarker for the diagnosis of GC.	[40]
circRNA_100269	It is an independent predictor of early recurrence of stage III GC. Its role in cancer progression remains unknown.	Down-regulated	–	Nanfang Hospital of Southern Medical University	December 2012 to May 2015	–	It is negatively correlated with miR-630; both of them comprise a novel pathway that regulates the proliferation of GC cells.	[41]
circLARP4	It is derived from the LARP4 gene locus.	Down-regulated	–	Downloaded from the Cancer Genome Atlas 2015 RNA sequencing database	–	–	It may act as a novel tumour suppressive factor and is a potential biomarker for GC.	[42]
hsa_circ_0014717	The global expression profile of this circRNAs in human GC has not yet been revealed. It is one of the moderately down-regulated circRNAs in microarray screening results.	Down-regulated	5	Affiliated Hospital of Medical School of Ningbo University (China)	February 2011 to February 2016	–	It has the potential to be used as a novel biomarker for the screening of high-risk GC patients.	[43]
hsa_circ_0000026	Its expression was significantly different between the GC and control samples (*P* = 0.001) in both qPCR and microarray analyses.	Down-regulated	≥5	Affiliated Hospital of Hainan Medical University (Haikou, China)	June 2014 to July 2014	2.8	It can regulate RNA transcription, RNA metabolism, gene expression, and gene silencing, and it also has other biological functions.	[44]
hsa_circ_0000745	It is down-regulated in GC tissues compared to non-tumorous tissues and in plasma samples from patients with GC vs healthy controls.	Down-regulated	–	Hospital Affiliated to Anhui Medical University (China)	January 2016 to January 2017	–	It plays an important role in GC, and its expression level in plasma can be measured in combination with the CEA level.	[45]
circPVT1	It is derived from the PVT1 gene locus and is frequently upregulated in patients with GC.	Up-regulated	–	Fudan University, Shanghai Cancer Center (FUSCC)	December 2007 to December 2010	–	It is a novel proliferative factor and prognostic marker in GC.	[46]
Hsa_circ_002059	It is one of the circRNAs associated with GC according to bioinformatics analysis in two circRNA databases: CircBase and circ2Traits.	Down-regulated	5	Yinzhou People’s Hospital and the Affiliated Hospital of Ningbo University, China	June 2012 to December 2013	–	It may be a potential novel, stable biomarker for the diagnosis of GC.	[47]
hsa_circ_0001895	It may be associated with GC according to the bioinformatics analysis in CircBase database.	Down-regulated	5	Affiliated Hospital of Ningbo University School of Medicine, China	November 2014 to February 2016	–	It may play crucial roles in GC initiation and it is a potential biomarker for prognosis prediction.	[48]
hsa_circ_0000520	It significantly down-regulated based on the microarray findings.	Down-regulated	5	Nanjing Hospital, affiliated with the Nanjing Medical University, China	2015–2016	–	It could serve as a novel biomarker for GC, and it is involved in GC development.	[49]

So, it is highly necessary to study the circRNAs’ category, quantity, location and functions in gastric cancer as soon as possible.

## Method summary

### Get the tissues

First, we need to determine our study design, object, type I error (a), statistical power (1-beta), related indicators and laboratory funding to determine our sample size [52]. For example, our study design is the two-sample paired t test, a is 0.05, beta is 0.1, mean of the paired difference is 2 and the standard deviation is 5, 10 and 15 respectively. We can get the paired sample size of 68, 265 and 593 respectively using PASS 11 software. Meanwhile we should acquire the samples with the approval of the Clinical Research Ethics Committee.

### Detect the different expression profiles in cancer cells and normal cells

RNA-Seq and gene chip have become common and preferred methods. There are several computational tools that have been developed to further identify circRNAs [53], including CIRCexplorer [29], find_circ [24], CIRI [54], KNIFE [55], NCLscan [56], DCC [57] and UROBORUS [58]. Thousands of circRNAs have been identified from RNA-Seq and gene chip using these computational tools, in humans, animals and plants. With these methods we can find the differential circRNA expression profiles between the cancerous and normal tissues and give annotation to the selected circRNA. Then one can pay attention to the greatest differential candidate and compare it with the current online circRNA databases or authoritative study results with Venn diagram [59].

### Confirm candidate circRNAs

The difference between circRNA and linear RNA lies in the structure (circRNA is circular), location and content. Firstly, we could confirm its circular structure. Generally we use divergent primers and convergent primers to verify its circular formation also with RNase. The circular junction can be confirmed by Sanger sequencing, which is based on the nucleotide at a fixed point, at random at a particular base of the termination, and after each base of the fluorescent marker, produced with a, T, C, G end of a series of four different lengths of nucleotide, and then on the urea degeneration of the PAGE gel electrophoresis test. FISH analysis can be used to demonstrate the location of the candidates, which is an important nonradioactive in situ hybridization technique, using the immune chemical reaction between the reporter molecules such as biotin and digoxin on a nucleic acid probe, but also qRT-PCR.

### Evaluate the function of circRNAs

When we talk about the function of circRNA, we first think about whether it follows the central principle and has the function of encoding proteins like mRNA. We have already verified its structure with above methods. Next, we should explore whether the circRNA has the ability to encode protein. We know circRNA does not have a 5′ end structure and the internal ribosome entry site (IRES) is required for 5′-cap-independent translation. So, if we want to clarify that circRNA has the ability to encode, we should confirm it has IRES. Now, we usually use a dual-luciferase vector system to confirm whether it has putative IRES activity, [60] with a set of vectors translated in human cells. If the circRNA can encode protein, we can use high performance liquid chromatography (HPLC) or western blotting (WB) to verify the amino acid sequences of the coded substance.

As we mentioned above, circRNAs can be used as molecular sponges for miRNA, and also have a relation with some proteins. In a comprehensive review of the relevant research studies, nearly all of the studies identified that the miRNAs interacted with circRNAs firstly through a related website, such as Target scan and the circular RNA interactome. After we know the interacted miRNA and protein, RNA Immune Precipitation (RIP) reaction and luciferase reporter gene assay are used to further confirm the relation.

We have to pay attention to the function of circRNA in cancers, for example gastric cancer. The most commonly used animal model is nude mouse to perform in vivo experiments. It is a mutant mouse with congenital thymus defects, lacking an immune response. Under certain circumstances, the nude mouse does not repel tissue transplants from heterogeneous animals. Therefore, it is commonly used as the recipient of transplanted human tumors. EDU, CCK8 and cell trans-well assay and so on are used to perform in vitro studies.

### Explore the molecular mechanism

Currently, the mechanism of the effect of circRNA on the tumor is still unclear. To sum up, the mechanism of mostly reports involved circRNA-miRNA-mRNA, circRNA-miRNA-protein and circRNA-miRNA-pathway interactive networks [61]. There are also studies of researchers on the relationship between circRNA and parent genes [30]. The method is similar to that mentioned above.

When it comes to mechanisms, it is inevitable to talk about circRNA’s biogenesis. As we mentioned above, circRNAs can mainly be classified in three categories: exon-exon circRNA (ecircRNA), exon-intron circRNA (EIcirRNA) and intron-intron circRNA (icircRNA). Intron pairing [7], snRNAPs (small nuclear ribonucleoproteins) [62] and RBPs [40, 63] pairing mainly form ecircRNA and EIcirRNA. Meanwhile lariat structure can form all of them [30]. The details can be seen in the two reviews by Esther Arnaiz et al. [64] and Bing Han et al. [65]. But it is still largely unknown about the mechanism of circRNA; more attention should be paid to it.

### Study of its biomarker effect

What is a biomarker? The Biomarkers Definitions Working Group proposed a definition of biomarker to describe the biological measurements in therapeutic development and assessment: “a characteristic that is objectively measured and evaluated as an indicator of normal biological processes, pathogenic processes, or pharmacologic responses to a therapeutic intervention” [66]. So, the diagnosis and prognosis of clinical trials are all needed to confirm the circRNAs’ biomarker effect.

Many researchers now determine whether a circRNA can be used as a biomarker for the diagnosis and prognosis of gastric cancer by detecting the difference in the expression in gastric cancer tissues and normal tissues or plasma with the area under the ROC (receiver operating characteristic) curve, that is to say clinical trials. The closer the AUC is to 1, the better the diagnostic effect is. AUC has lower accuracy when 0.5–0.7, certain accuracy when 0.7–0.9, and higher accuracy when above 0.9. Equal to 0.5, it is indicated that the diagnostic method is completely ineffective and has no diagnostic value. Less than 0.5 indicates that it does not conform to the real situation and rarely appears in practice. Therefore, we need to ensure the credibility of the patient data we collected for later exploration experiments.

**Fig. 5:**
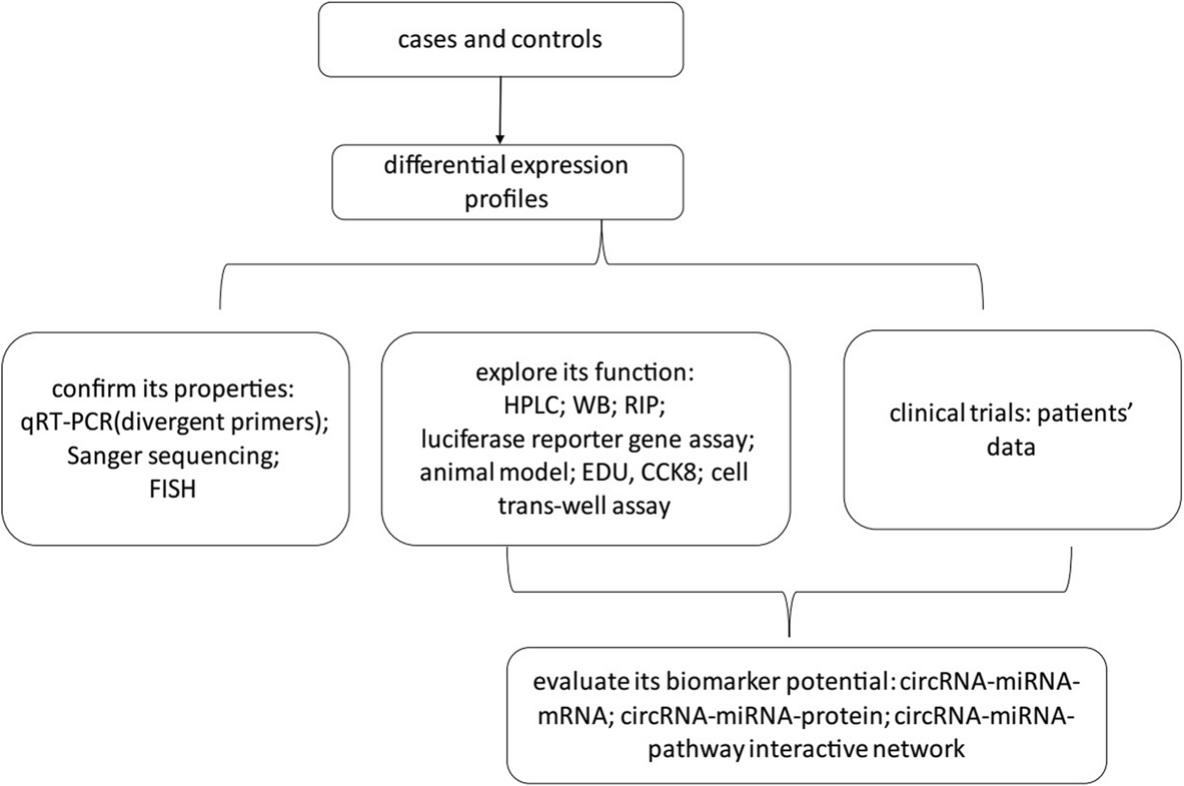
Methods for studying the properties, functions, and mechanisms of circRNAs and for determining their potential use as biomarkers

To sum up, the research on circRNA is mainly divided into three directions: functional research, molecular mechanism study and clinical direction as biomarker. The research methods mentioned above are shown in Fig. 5.

## Discussion

Without doubt, gastric cancer puts lots of pressure on human life. Its epidemiology trend was affected by region, age and gender [67]. Although there are many new cases and death cases for gastric cancer every year, which are not evenly distributed around the world, more than half of all cases occur in Eastern Asia, Central and Eastern Europe, and Central and South America [1], which may be related to the prevalence of *H. pylori*. A systematic review reported the prevalence rates of *H. pylori* among different countries were: 71.4% in China (35–64 years, 1989); 23% in Hungary (19–23 years, 1999–2000); 33% in Iceland [median age ± standard deviation (SD): 27 ± 0.3 years, 1975–1997]; 46.5% in Israel (mean age ± SD: 18.73 ± 0.74 years, 1986–1995); 52.0% in Lebanon (mean age ± SD: 40.97 ± 15 years, 2008–2009); 50.2% in Singapore (55–69 years, 1998); 51% in San Marino (20–79 years, 1990–1991); and 13.4% in the United Kingdom (1–84 years, 1986–1996) [68]. We can hypothesize that the prevalence of *H. pylori* affects the gastric cancer distribution. Meanwhile, there are even variants of CagA in East Asian strains that may further increase the risk of gastric cancer [69]. So, it is necessary to stratify by country or region to obtain the most authentic and dependent evidence to understand and treat gastric cancer. As we all know, the incidence and mortality of gastric cancer in males are both nearly 2 times higher than in females, wherever [67]. We also know that the high level of salt, low fruit intake, alcohol consumption and active tobacco smoking all are established risk factors [70], while, generally speaking, males are less concerned about diet control and tend to consume tobacco and alcohol. So we inferred that these factors contribute to gastric cancer affecting males much more than females. It also indicates that we can stratify gastric cancer patients not only by gender but also by dietary habits or tobacco and alcohol intake. Meanwhile, we cannot ignore that gastric cancer has a predilection towards the elderly population, which may be related to the incidence of pre-neoplastic gastric lesions in parallel with increased age [71]. The median age of diagnosis for gastric cancer in males and females is 71 and 68 years, respectively, in Hong Kong [72]. To sum up, if we want to have a proper understanding about gastric cancer, we need to build a sense of layered analysis when analyzing its trends, morbidity or mortality.

However, most important, we should know how to prevent, diagnose and treat gastric cancer.

We have to admit that despite CA199, CEA, and CA724, these tumor biomarkers are used in the detection of gastric cancer, but with low sensitivity and specificity [73]. Some researchers have also been reporting molecular microRNAs as a diagnostic and therapeutic biomarkers in gastric cancer, such as mi-21 [74], miR-378 [75] and so on, which is in the exploratory phase, now. We can find that current diagnostic and therapy methods are not satisfactory.

As we mentioned before, circRNAs have several remarkable characteristics as biomarkers. Firstly, circRNAs are abundant in the human body. Numerous examples of transcripts in which the exon order was a circular permutation in both cancer and normal human cells were found by Julia Salzman et al. [25]. Secondly, they are stable. circRNAs have covalently closed loop structures, lacking 5′-caps end and 3′-polyA tails, which results in higher stability than linear RNAs, in RNase for example. Then, the expression of them is specific, especially in the disease development stage. What is more, circRNAs can be detected not only in tissues but also in the exome, blood and saliva. Exosomes are membrane vesicles that can be released into the extracellular environment upon exocytic fusion of multivesicular endosomes with the cell surface [76], containing a specific cargo of protein, mRNA and miRNA species. Yan Li et al. first reported the presence of abundant circRNAs in exosomes. Additionally, this study team identified more than 1000 circRNAs in human serum exosomes, which suggests that circRNAs can be considered as a new class of exosome-based cancer biomarkers. However, Weiwei Tang et al. found that circ-KIAA1244 is obviously down-regulated in GC tissues, cells, and plasmas compared to normal controls but exosomes [77]. Based on these results, we proposed that circRNAs are encapsulated in the exosomes, which can be decomposed by the large amount of RNase present in plasma. There is also an inevitable error in the operation of the experiment. Tianwen Li et al. found 343 differentially expressed circRNAs between gastric cancer patients’ plasma and healthy controls by circRNA microarray [78]. The study of circRNAs as biomarkers in plasma has not only been discussed in gastric cancer, but has also been studied in other diseases. For example, Zhang YG et al. found that circ_101222 in blood corpuscles combined with plasma protein factor strengthened the predictive power for pre-eclampsia [79], Nicolet BP et al. reported that circRNA in hematopoietic cells has a cell-type specific expression pattern [80], which again suggests that circRNA has a huge potential function throughout the whole life process.

It has been demonstrated that circRNAs can regulate the growth, apoptosis, and cell cycle progression of tumor cells, including colon cancer [81], gastric cancer [82], heart failure [83], hepatocellular carcinoma [84], glioma tumorigenesis [32] and so on. We cannot ignore that circRNAs in other diseases have also been reported, such as immune system diseases [85], and even diabetes [86], one of the crucial health issues worldwide.

However, we are still confused about the function of circRNAs in the life process despite the vast experiments performed. First of all, we should conduct in-depth research on the biogenesis of circRNA, which can make the mechanism clear and lay the basis for classification of circRNA. Then, we may find that circRNA is a suitable biomarker and a good strategy in therapy in many diseases, but how to convert it into medicine is still a problem for researchers and doctors. Lastly, we have to say the normativity of the operation and the quality of samples used in related experiments are uncontrollable, while they affect the results directly. Thus far, we believe that with the progress of research, we will have a deeper understanding of circRNA.

## Conclusion

In summary, circRNAs provide new insights into the ‘dark matter’ of the human genome [59]. In present studies we can find that circRNA is a hot topic, and its function as an effective biomarker in diagnostics and prognosis for gastric cancer or other diseases is obvious and shocking us. However, circRNA still has many unknown features waiting for us to continue in-depth studies to reduce human suffering and improve human living standards. In addition, this review also introduces the laboratory methods; in fact, making the results of study have a greater application in clinical practice is more important, which is what our team will next focus on.

## Additional files

Additional file 1: **Figure S1.** Distribution of gastric cancer incidence and mortality worldwide; the place with red spots indicates high incidence and mortality of gastric cancer (from left to right: East Asia, Central and Eastern Europe and South America). (JPG 149 kb)

Additional file 2: **Figure S2.** Distribution of gastric cancer incidence and mortality in China; the place with red spots indicates high incidence and mortality of gastric cancer (south and east of China). (JPG 300 kb)

## Data Availability

The data in this study are available from the author for correspondence upon reasonable request.

## Abbreviations

circRNA: Circular RNA
GC: Gastric cancer
miRNA: MicroRNA
