# Supplementary figures and images for: Current prevalence status of gastric cancer and recent studies on the roles of circular RNAs and methods used to investigate circular RNAs
Source: Cell Mol Biol Lett. 2019 Aug 16;24:53. doi: 10.1186/s11658-019-0178-5 (PMC6698018; doi:10.1186/s11658-019-0178-5)

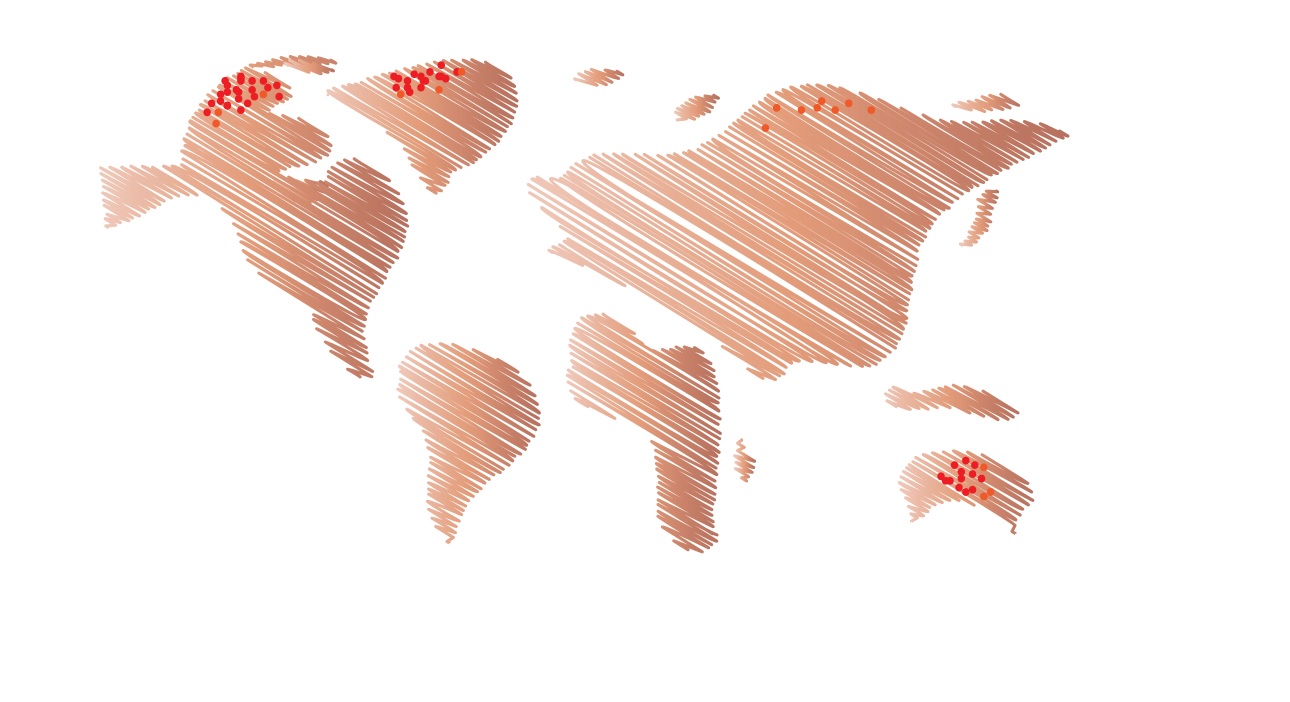

Supplement: Supplementary file 1 — Figure S1. Distribution of gastric cancer incidence and mortality worldwide; the place with red spots indicates high incidence and mortality of gastric cancer (from left to right: East Asia, Central and Eastern Europe and South America). (JPG 149 kb) [file 11658_2019_178_MOESM1_ESM.jpg]

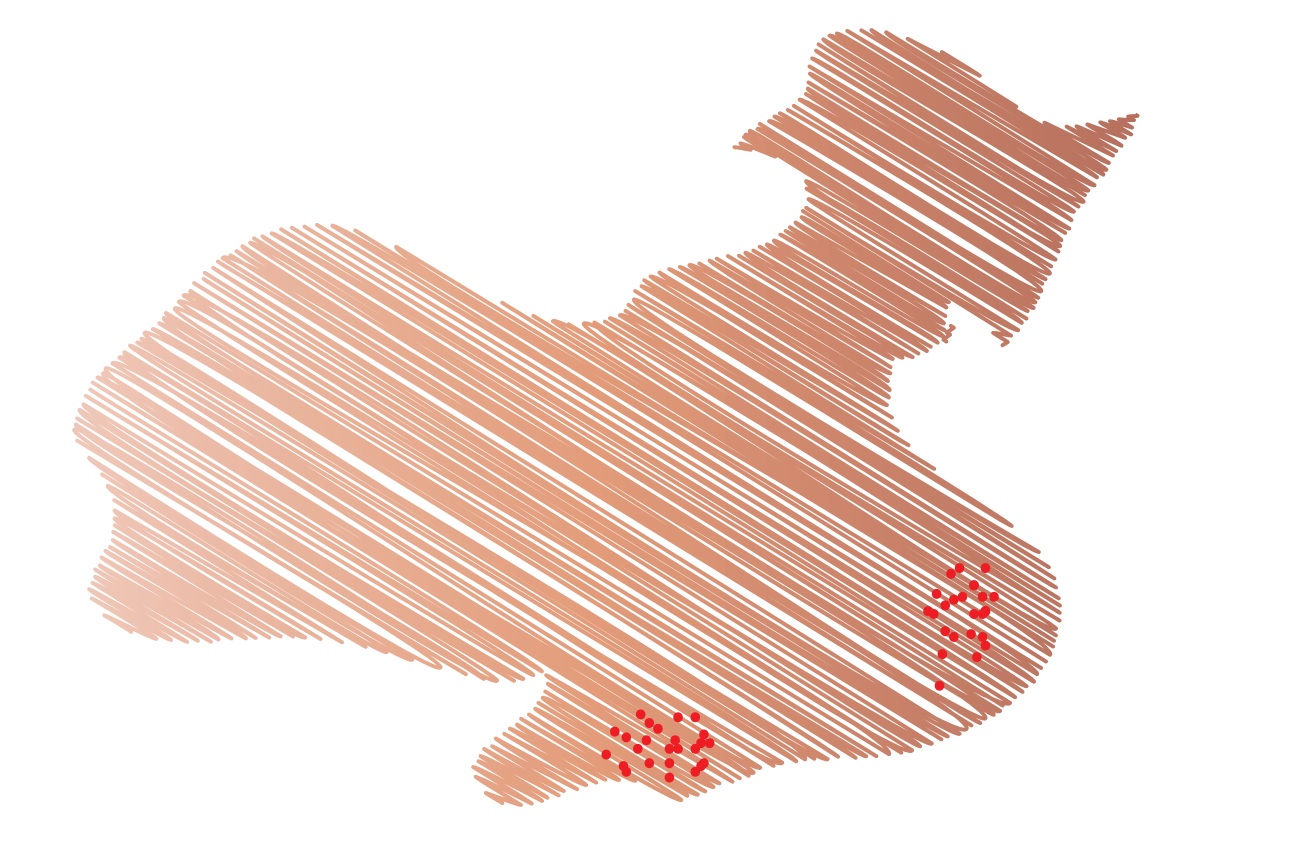

Supplement: Supplementary file 2 — Figure S2. Distribution of gastric cancer incidence and mortality in China; the place with red spots indicates high incidence and mortality of gastric cancer (south and east of China). (JPG 300 kb) [file 11658_2019_178_MOESM2_ESM.jpg]
